# Bimodal Patterning Discrimination in Harnessed Honey Bees

**DOI:** 10.3389/fpsyg.2018.01529

**Published:** 2018-08-24

**Authors:** Breno E. Mansur, Jean R. V. Rodrigues, Theo Mota

**Affiliations:** Department of Physiology and Biophysics, Federal University of Minas Gerais, Belo Horizonte, Brazil

**Keywords:** classical conditioning, bimodal learning, negative patterning, positive patterning, inter-trial interval, insect, honey bee

## Abstract

In natural environments, stimuli and events learned by animals usually occur in a combination of more than one sensory modality. An important problem in experimental psychology has been thus to understand how organisms learn about multimodal compounds and how they discriminate this compounds from their unimodal constituents. Here we tested the ability of honey bees to learn bimodal patterning discriminations in which a visual-olfactory compound (AB) should be differentiated from its visual (A) and olfactory (B) elements. We found that harnessed bees trained in classical conditioning of the proboscis extension reflex (PER) are able to solve bimodal positive and negative patterning (NP) tasks. In positive patterning (PP), bees learned to respond significantly more to a bimodal reinforced compound (AB+) than to non-reinforced presentations of single visual (A-) or olfactory (B-) elements. In NP, bees learned to suppress their responses to a non-reinforced compound (AB-) and increase their responses to reinforced presentations of visual (A+) or olfactory (B+) elements alone. We compared the effect of two different inter-trial intervals (ITI) in our conditioning approaches. Whereas an ITI of 8 min allowed solving both PP and NP, only PP could be solved with a shorter ITI of 3 min. In all successful cases of bimodal PP and NP, bees were still able to discriminate between reinforced and non-reinforced stimuli in memory tests performed one hour after conditioning. The analysis of individual performances in PP and NP revealed that different learning strategies emerged in distinct individuals. Both in PP and NP, high levels of generalization were found between elements and compound at the individual level, suggesting a similar difficulty for bees to solve these bimodal patterning tasks. We discuss our results in light of elemental and configural learning theories that may support the strategies adopted by honey bees to solve bimodal PP or NP discriminations.

## Introduction

Living in a complex world demands learning and memory of relationships between diverse stimuli in the environment. Animals can learn to associate an originally neutral stimulus (conditioned stimulus, CS) with a meaningful stimulus (unconditioned stimulus, US), an elemental association that constitutes the basis of classical conditioning (Pavlov, 1927). However, natural environments are composed by multimodal stimuli and animals usually associate these compounds with an US, rather than single unimodal elements (Lorenz, 1951). For instance, honey bees *Apis mellifera* use their learning capacity to exploit food sources in flowers displaying multimodal signals like colors, shapes and odors (Menzel and Mercer, 2012). Although several studies have shown that honey bees can learn to associate a single odor or color with sucrose reward (Sandoz, 2011; Menzel and Mercer, 2012), little is known about how they combine or filter relevant stimuli of distinct sensory modalities during multimodal learning tasks (Leonard and Masek, 2014).

Multimodal appetitive learning has been mostly studied using operant conditioning of free-flying bees, but results obtained so far gave rise to different conclusions. On the one hand, several studies indicated a synergistic effect between color and odor within a bimodal compound, so that combined color-odor cues led to better learning and memory compared with unimodal cues (Kunze and Gumbert, 2001; Reinhard et al., 2004, 2006; Kulahci et al., 2008). On the other hand, other studies reported inhibitory effects within a color-odor compound, so that odors tend to overshadow colors based on differences in salience (Couvillon and Bitterman, 1982, 1988, 1989; Couvillon et al., 1983; Funayama et al., 1995; Greggers and Mauelshagen, 1997). An important limitation of studies on multimodal learning in free-flying bees could be the reason of these contradictory results: differences in temporal characteristics of the two stimuli. When bees approach a color-odor cued feeder or Y-maze, color may act as a far-distance signal, and odor as a close-up signal. It is thus difficult to interpret bees’ performance, given that sequential rather than simultaneous stimulus processing may occur during the approach to the target (Mota et al., 2011). These two scenarios, sequential versus simultaneous stimulus processing, may determine dramatic differences in performance, such as those supporting synergistic versus inhibitory within-compound processing.

Classical conditioning of the proboscis extension reflex (PER) in harnessed bees represents a promising alternative to study bimodal appetitive learning with a precise control of stimuli timing and duration (Gerber and Smith, 1998; Mota et al., 2011; Hussaini and Menzel, 2013). Nevertheless, such experiments are so far rare, probably because of the difficulty of training harnessed bees with visual cues (Avarguès-Weber and Mota, 2016). Whereas PER conditioning has been used for more than 50 years to study olfactory learning and memory in bees (Sandoz, 2011), only in the last two decades successful visual-PER conditioning has been achieved (Avarguès-Weber and Mota, 2016; Vieira et al., 2018). In the present work, we took advantage of this classical conditioning protocol to study bimodal patterning learning in harnessed honey bees.

Solving of patterning discriminations is considered a higher-order form of associative learning, because it involves non-linearity and intrinsic stimulus ambiguity (Rudy and Sutherland, 1995; Giurfa, 2003). In positive patterning (PP), animals have to differentiate a reinforced compound stimulus AB+ from its non-reinforced single elements A- and B-. In negative patterning (NP), single elements A+ and B+ are reinforced whereas the compound AB- is non-reinforced (Pavlov, 1927). The non-linearity of these patterning tasks resides in the fact that the contingency of the compound AB cannot be predicted by the simple linear summation of the contingencies of the single elements (A and B). Under these conditions, associative learning also implicates relational dependencies, as the contingencies of a given stimulus (e.g., A) vary as a function of its occurrence alone or in combination with other stimuli (stimulus ambiguity). Therefore, these patterning tasks tend to require *configural learning*, i.e., the ability to treat the compound stimulus as different from the simple sum of its elements (Giurfa, 2003). Whereas NP could only be solved through *configural learning*, PP may also be accomplished through *elemental learning*. According to the elemental summation principle, the associative strength of each single element in a PP task could be subthreshold for the response, but the threshold could be exceeded when both elements are combined in a compound. In NP, however, the sum of the excitatory strengths of the elements in a compound will always be higher than the strength of each single element (Deisig et al., 2001; Pearce and Bouton, 2001).

The honey bee is the only insect model that, as mammals, was shown to have the ability of solving both PP and NP tasks (Devaud et al., 2015). Studies in flies and bumble bees found that these insects can solve PP, but not NP tasks, thus suggesting their inability to accomplish *configural learning* problems (Young et al., 2011; Sommerlandt et al., 2014). Learning of PP and NP by honey bees has been traditionally studied using olfactory conditioning of the PER (Chandra and Smith, 1998; Deisig et al., 2001, 2002, 2007; Komischke et al., 2003; Devaud et al., 2015). The capacity to solve PP and NP was also demonstrated in free-flying honeybees trained to visual stimuli in an operant framework (Schubert et al., 2002). No study has so far analyzed in a well-controlled way the capacity of insects to solve patterning discriminations using stimuli of distinct sensory modalities, as traditionally performed in rats and rabbits (Whitlow and Wagner, 1972; Bellingham et al., 1985; Kehoe and Graham, 1988). To our knowledge, the only attempt of studying bimodal patterning learning in an insect model was made by Couvillon and Bitterman (1988). Nevertheless, these authors trained free-flying honey bees using visual and olfactory stimuli that presented distinct detection ranges and were thus perceived in a sequential way by bees (Deisig et al., 2001; Mota et al., 2011). Here, we fill this gap by training honey bees to bimodal PP and NP using equivalent duration of all stimuli, as well as simultaneous (not sequential) presentation of visual (A) and olfactory (B) elements in a compound (AB).

Previous studies showed that the temporal separation between stimuli trials (intertrial interval - ITI) clearly affects the learning performance of honey bees in olfactory patterning discrimination. Both in PP and NP tasks, increasing the ITI between conditioned trials led to better differentiation between single olfactory elements and their compound mixture (Deisig et al., 2007). So in the present study, we compare the performance of honeybees in bimodal PP and NP tasks using a shorter or a longer ITI. We also analyze memory retention to each unimodal element and the bimodal compound one hour after conditioning. Furthermore, we evaluate differences in the individual learning and memory performances of bees during these bimodal patterning tasks. Our work represents an important step to uncover the cognitive and neurobiological basis of bimodal patterning discriminations in insects.

## Materials and Methods

### Animals

Foragers of honeybee *A. mellifera* were collected from a feeder containing 30% (v/v) sugar solution 50 meters from six outdoor hives kept in the Ecological Station of the Federal University of Minas Gerais (UFMG, Brazil). All experiments were conducted in the Brazilian spring/summer season (from September to March). Bees were placed in small glass vials, cooled on ice until they ceased their movements and then harnessed in plastic tubes using thin pieces of soft masking tape. The wings were protected by a piece of filter paper. Each bee was fed 1 μl of 30% (v/v) sugar solution after fixation and then kept for one hour in a dark chamber with high humidity.

### Conditioned and Unconditioned Stimuli

Visual CS (A) consisted of an illuminated 20 × 20 cm screen covered with a chromatic transmission filter (LF124S *Dark Green*: peak at 535 nm or LF119S *Dark Blue*: peak at 455 nm; LEE Filters) and tracing paper for light dispersion. A white-LED light source (E27-5W *Cool White*; Epistar) connected to a linear potentiometer provided illumination with controlled intensity behind the colored screen. Taking into account the spectral sensitivities of the honeybee photoreceptors (Peitsch et al., 1992), the green stimulus excited 0, 15, and 85% of the short- (S), medium- (M), and large-range (L) wavelength photoreceptors, respectively. For the blue stimulus, these values were 2, 68, and 30%, respectively. The large-field colored screen was placed at a distance of 10 cm from the bee right eye, so that it subtended a visual angle of 90°. The irradiance of blue or green stimulus was adjusted to 0,4 μW cm^-2^ at the level of the bee eye by using a spectrophotometer (USB2000 + UV-VIS-ES, Ocean Optics) radiometrically calibrated using a deuterium/tungsten light source (DH-2000-BAL, 220-1050 nm, Ocean Optics). Absolute irradiance was measured using an optical fiber (QP600-2-UV-VIS, Ocean Optics) coupled to a cosine corrector with Spectralon diffusing material (CC-3-UV-S, Ocean Optics).

Olfactory CS (B) was 2-hexanol or 1-nonanol (Sigma-Aldrich, Brazil). Five microliters of pure odorant were applied onto a 1 cm^2^ stripe of filter paper placed into a 30 mL syringe, which allowed frontal odorant delivery to the antennae. An air extractor placed behind the bee prevented odorant accumulation.

The US was 1 μL of 30% (w/w) sugar solution delivered to the bee by means of a micropipette.

The reason we presented a lateral screen stimulating a single eye instead of a frontal one stimulating both eyes was the fact that both the syringe used to deliver the odor and the micropipette used to deliver sugar solution were already presented in a frontal position. When odor, color and reward overlapped during patterning conditioning, the syringe and the micropipette produced large shades on the visual screen. These shades may be used by the bees as conditioned or secondary stimulus. We thus decided to present visual stimulation only to the right eye, because a previous study on visual conditioning of the PER indicates that honey bees learn better in this framework using the right than the left eye (Letzkus et al., 2008). After this work, other authors confirmed that lateral stimulation of the right eye is an efficient method for training harnessed bees to visual stimuli (e.g., Niggebrügge et al., 2009; Vieira et al., 2018).

### Experimental Setup and Conditioning Procedure

All experiments were performed in a dark room illuminated by a low intensity red-light source (peak at 660 nm). During conditioning, the plastic tube holding the bee was tilted to 45° and fixed in a platform of 9 cm high (Vieira et al., 2018). In this position, the right eye of the bee was at a distance of 10 cm from the center of the visual stimulation screen. In PP experiments, presentation of visual or olfactory stimulus alone was not rewarded whereas their simultaneous presentation (compound stimulus) was rewarded (A-, B-, and AB+). In NP experiments, individual presentations of the visual or olfactory stimulus were rewarded whereas the compound bimodal stimulus was not (A+, B+, and AB-). Both in PP and NP, training consisted of 10 trials of each stimulus (A, B, and AB), thus totalizing 30 trials presented in a pseudorandom sequence starting with A, B or AB in a balanced way. At most, two trials of a same stimulus followed each other during conditioning.

At the beginning of each rewarded trial the bee was placed in the conditioning setup for 30 s to allow familiarization with the experimental context. Thereafter, CS+ (A, B or AB) was presented for 7 s. Four seconds after the onset of the CS+, the US was delivered to the bee for 3 s. Therefore, the interstimulus interval (ISI) was 4 s and the overlap between CS and US was 3 s. The bee was removed from the setup 23 s after reward, thus completing a total of 60 s per trial. Unrewarded trials followed the same time sequence, but stimulation was not paired with reward. To analyze the effect of inter-trial interval (ITI) on bimodal PP and NP, we trained two independent groups in each of these paradigms with an ITI of 3 and 8 min, respectively. Training with 3 min ITI was performed using green 535 nm as visual stimulus and 2-hexanol as olfactory stimulus (*N* = 45 bees, both for PP and NP). Training with 8 min ITI was performed using this same pair of stimuli (green 535 nm and 2-hexanol) or an alternative pair consisted of blue 455 nm and 1-nonanol (*N* = 45 bees/pair of stimuli, both for PP, and NP). One hour after the end of conditioning, all experimental groups were submitted to retention tests consisted of an unrewarded presentation of each stimulus (A, B, or AB) with an ITI equivalent to that used during training (3 min or 8 min ITI). The order of presentation of the three stimuli during retention tests was randomized between subjects in all experimental groups.

The beginning and the end of each trial, as well as the onset and offset of CS and US were signaled by a computer programmed to emit tones of different frequencies for each event. The occurrence of proboscis extension was recorded within the first 4 s of CS presentation (conditioned response), as well as during the US presentation. Animals that did not show PER for more than 3 times during the US presentation (<5%) were excluded from our analysis, as they may present impairment of muscular reflex and/or sucrose responsiveness.

### Statistical Analysis

Two-way analysis of variance in generalized linear model (GLM) for repeated measures was used to analyze within (*stimulus* × *trial* effect) and between group (*group* × *stimulus* × *trial* effect) performances in PP and NP conditioning. Further Tukey’s multiple comparisons were used to analyze differences between: (i) responses to each stimulus; (ii) performances in different trials of conditioning. One-way GLM for repeated measures followed by Tukey’s multiple comparisons was used to compare responses to each stimulus in retention tests. The alpha level was set to 0.05 (two tailed) for all analyses. All statistical analyses were conducted using the software IBM SPSS Statistics 21.0.

## Results

### Bimodal Patterning Discrimination With 3 min ITI

**FIGURE 1:**
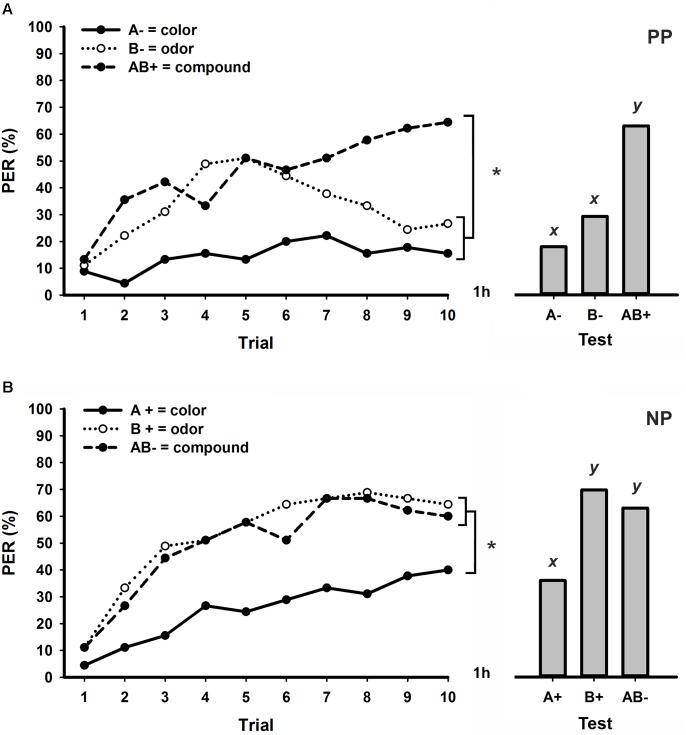
Responses (% PER) of honey bees to the visual element (green 535 nm), the olfactory element (2-hexanol) and the bimodal compound during patterning discrimination tasks with an ITI of 3 min. Conditioning consisted of 10 trials of each stimulus presented in a pseudorandom sequence (left). One hour after conditioning, unrewarded retention tests were performed for each stimulus (right). **(A)** Positive patterning (PP; *N* = 45). **(B)** Negative patterning (NP; *N* = 45). Asterisks indicate significant differences in GLM followed by Tukey test comparing responses to each stimulus in the last conditioning trial. Different lowercase letters (*x,y*) indicate significant differences in GLM followed by Tukey test comparing responses to each stimulus during retention tests.

Honeybees trained to discriminate a visual element (A-) and an olfactory element (B-) from its bimodal compound (AB+) in a PP protocol with an ITI of 3 min were successful in learning the task. **Figure 1A** shows the percentage of PER along 10 trials of each stimulus and reveals significant differences between the learning curves of A-, B-, and AB+ (*stimulus* × *trial* GLM for repeated measures; *stimulus effect*: *F*_2,88_ = 19.6, *p* < 0.001; *interaction: F*_18,792_ = 5.4, *p* < 0.001). During the first five trials, bees showed equivalent levels of increasing response to the olfactory element (B-) and the compound (AB+) whereas responses to the visual element (A-) remained much lower. After the fifth trial, however, responses to B- started to decrease whereas responses to AB+ kept increasing until the end of conditioning (**Figure 1A**). Although global performances significantly differed between all stimuli (Tukey test; *stimulus effect*; A- vs. B- and A- vs. AB+: *p* < 0.001; B- vs. AB+: *p* < 0.05), no differences were found in responses to A- and B- at the last trial of conditioning (Tukey test; *stimulus* × *trial 10 effect*; A- vs. B-: NS). Moreover, bees responded significantly more in the last trial to the compound AB+ than to the elements A- and B- (Tukey test; *stimulus* × *trial 10 effect*; A- vs. AB+ and B- vs. AB+: *p* < 0.001), thus confirming successful PP solving. In retention tests performed one hour after conditioning (**Figure 1A**), bees were again able to discriminate each unimodal element from the bimodal compound (GLM for repeated measures; *F*_2,88_ = 20.2, *p* < 0.001; Tukey test; A- vs. B-: NS; A- vs. AB+ and B- vs. AB+: *p* < 0.001).

While honeybees were successful in learning a bimodal PP task with an ITI of 3 min (**Figure 1A**), this was not the case for a NP task with the same ITI (**Figure 1B**). In this patterning approach, bees showed increasing PER to all three stimuli along trials, with equivalent levels of response to the olfactory element B+ and the bimodal compound AB- (*stimulus* × *trial* GLM for repeated measures; *stimulus effect*: *F*_2,88_ = 25.9, *p* < 0.001; *interaction: F*_18,792_ = 1.8, *p* < 0.05; Tukey test; *stimulus effect*; A+ vs. B+ and A+ vs. AB-: *p* < 0.01; B+ vs. AB-: NS). The comparisons between the responses to each stimulus at the last conditioning trial also show that bees were unable to differentiate B+ from AB- in this NP task (Tukey test; *stimulus* × *trial 10 effect*; A+ vs. AB+ and A+ vs. AB- : *p* < 0.05; B+ vs. AB-: NS). Retention tests performed one hour after conditioning (**Figure 1B**) also confirm an absence of discrimination between the olfactory element and the bimodal compound (GLM for repeated measures; *F*_2,88_ = 11.0, *p* < 0.001; Tukey test; A+ vs. B+ and A+ vs. AB-: *p* < 0.05; B+ vs. AB-: NS).

### Bimodal Patterning Discrimination With 8 min ITI

**FIGURE 2:**
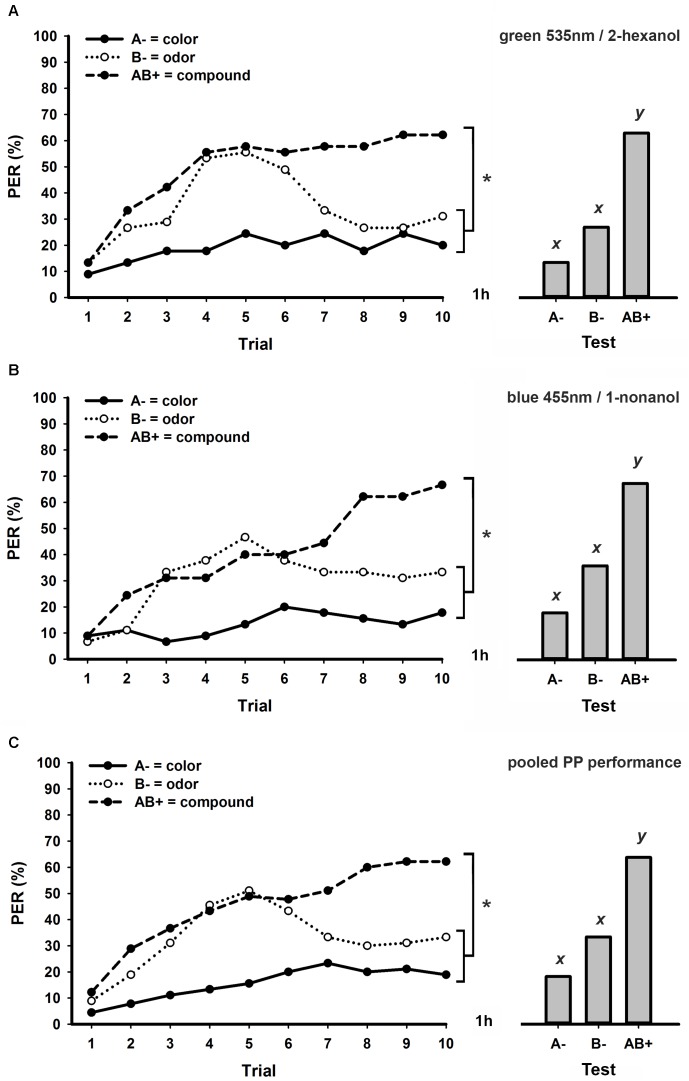
Learning curves (left) and responses during retention tests (right) of honey bees trained to a bimodal positive patterning (PP) task with an ITI of 8 min. **(A)** Experimental group conditioned using green 535 nm as visual element and 2-hexanol as olfactory element (*N* = 45). **(B)** Experimental group conditioned using blue 455 nm as visual element and 1-nonanol as olfactory element (*N* = 45). **(C)** Pooled performance of the two experimental groups (**A,B**; *N* = 90). Asterisks indicate significant differences in GLM followed by Tukey test comparing responses to each stimulus in the last conditioning trial. Different lowercase letters (*x,y*) indicate significant differences in GLM followed by Tukey test comparing responses to each stimulus during retention tests.

**Figure 2A** shows the performance of bees trained to a PP task using the same visual (green 535 nm) and olfactory stimuli (2-hexanol) as in **Figure 1A**, but with a longer ITI of 8 min. As previously observed (**Figure 1A**), bees started the task with similar increasing response levels to B- and AB+, but after the fifth trial they begun to discriminate these stimuli (*stimulus* × *trial* GLM for repeated measures; *stimulus effect*: *F*_2,88_ = 10.8, *p* < 0.001; *interaction: F*_18,792_ = 3.6, *p* < 0.001; Tukey test; *stimulus effect*: A- vs. B- and A- vs. AB+: *p* < 0.001; B- vs. AB+: *p* < 0.01). Responses at the last conditioning trial significantly differed between each unimodal element and the bimodal compound, but not between the visual and olfactory elements (Tukey test; *stimulus* × *trial 10 effect*; A- vs. B-: NS; A- vs. AB+ and B- vs. AB+: *p* < 0.001). Together with these results, responses of bees during retention tests (**Figure 2A**) confirmed successful bimodal PP solving (GLM for repeated measures; *F*_2,88_ = 20.6, *p* < 0.001; Tukey test; A- vs. B-: NS; A- vs. AB+ and B- vs. AB+: *p* < 0.001).

We simultaneously trained another group of bees to the same PP task with 8 min ITI, but using alternative visual (blue 455 nm) and olfactory stimuli (1-nonanol). The performance of bees in this experimental group (**Figure 2B**) was very similar to the one of bees trained using green 535 nm and 2-hexanol as conditioned stimuli (**Figure 2A**). They were able to discriminate each unrewarded unimodal element from the rewarded bimodal compound both during conditioning (*stimulus* × *trial* GLM for repeated measures; *stimulus effect*: *F*_2,88_ = 17.5, *p* < 0.001; *interaction: F*_18,792_ = 5.5, *p* < 0.001; Tukey test; *stimulus* × *trial 10 effect*; A- vs. B-: NS; A- vs. AB+ and B- vs. AB+: *p* < 0.001) and retention tests (GLM for repeated measures; *F*_2,88_ = 19.4, *p* < 0.001; Tukey test; A- vs. B-: NS; A- vs. AB+ and B- vs. AB+: *p* < 0.001). Since we found no statistical differences between these two experimental groups (**Figures 2A,B**) trained using distinct pairs of stimuli (*group* × *stimulus* × *trial* GLM for repeated measures; *group effect*: F_1,88_ = 1.3, NS), we pooled results from each of them in a single graphic (**Figure 2C**). As expected, all statistical effects in this pooled group (**Figure 2C**) were equivalent to those described for experimental groups presented in **Figures 1A,B** both during conditioning (*stimulus x trial interaction: F*_18,1584_ = 6.7, *p* < 0.001; Tukey test; *stimulus* × *trial 10 effect*; A- vs. B-: NS; A- vs. AB+. and B- vs. AB+: *p* < 0.001) and retention tests (GLM for repeated measures; *F*_2,178_ = 36.3, *p* < 0.001; Tukey test; A- vs. B-: NS; A- vs. AB+ and B- vs. AB+: *p* < 0.001).

**FIGURE 3:**
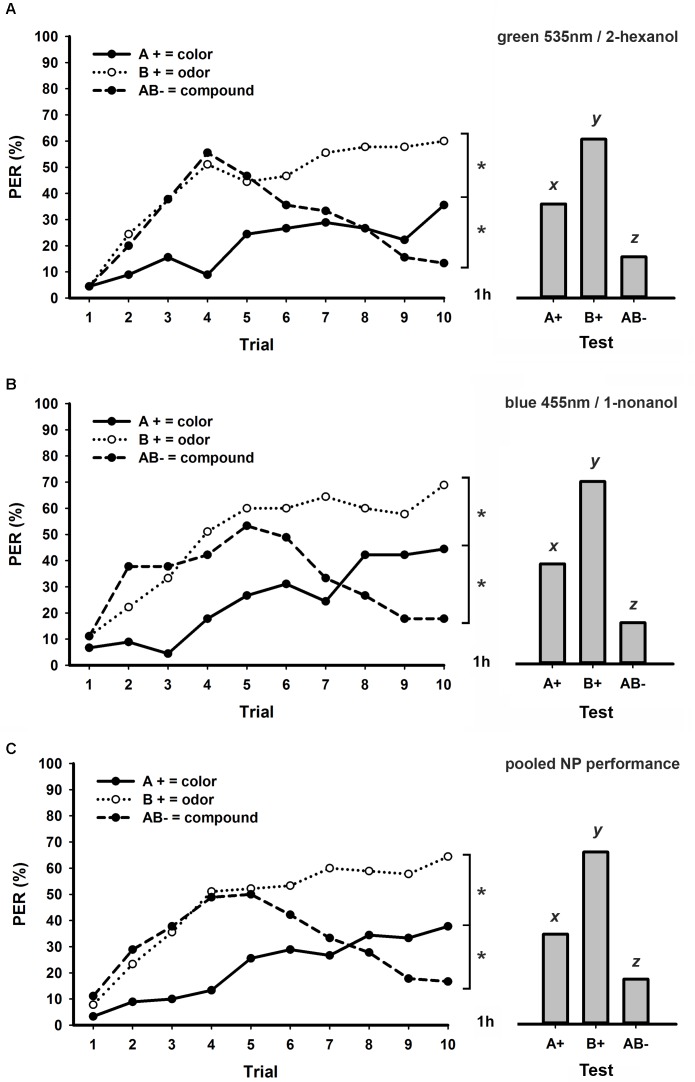
Learning curves (left) and responses during retention tests (right) of honey bees trained to a bimodal negative patterning (NP) task with an ITI of 8 min. **(A)** Experimental group conditioned using green 535 nm as visual element and 2-hexanol as olfactory element (*N* = 45 bees). **(B)** Experimental group conditioned using blue 455 nm as visual element and 1-nonanol as olfactory element (*N* = 45 bees). **(C)** Pooled performance of the two experimental groups (**A,B**; *N* = 90 bees). Asterisks indicate significant differences in GLM followed by Tukey test comparing responses to each stimulus in the last conditioning trial. Different lowercase letters (*x, y*, and *z*) indicate significant differences in GLM followed by Tukey test comparing responses to each stimulus during retention tests.

**Figure 3** shows the performances of honeybees trained in a bimodal NP task with 8 min ITI using green 535 nm and 2-hexanol (*group A*; **Figure 3A**) or blue and 1-nonanol (*group B*; **Figure 3B**) as pair of stimuli. Different from bees trained in a NP task with 3 min ITI (**Figure 1B**), we found that the larger ITI of 8 min allowed honeybees to solve a bimodal NP task. In both experimental groups (**Figures 3A,B**), bees started the task by increasing their responses to all three stimuli, but begun to decrease their levels of response to the unrewarded bimodal compound after the fourth or fifth trial. At the end of conditioning, both groups were able to discriminate each unimodal element from its bimodal compound (*stimulus* × *trial* GLM for repeated measures; *stimulus effect*; *group A*: *F*_2,88_ = 11.8, *p* < 0.001; *group B*: *F*_2,88_ = 16.7, *p* < 0.001; *interaction*; *group A*: *F*_18,792_ = 5.9, *p* < 0.001; *group B*: *F*_18,792_ = 6.7, *p* < 0.001; Tukey test; *stimulus* × *trial 10 effect*; *both groups*: A+ vs. AB-: *p* < 0.05; B+ vs. AB-: *p* < 0.001). Different from bees trained to bimodal PP tasks (**Figures 1A**, **2**), levels of response significantly differed at the end of conditioning between the visual and the olfactory elements (**Figures 3A,B**; Tukey test; *stimulus* × *trial 10 effect*; *both groups*: A+ vs. B+: *p* < 0.05). In retention tests performed one hour after conditioning (**Figures 3A,B**), levels of response were significantly different between all three stimuli (GLM for repeated measures; *group A*: *F*_2,88_ = 17.1, *p* < 0.001; *group B*: *F*_2,88_ = 23.2, *p* < 0.001; Tukey test; *both groups*: A+ vs. B+: *p* < 0.05; A+ vs. AB-: *p* < 0.05; B+ vs. AB-: *p* < 0.001).

We found no statistical differences between performances of these two experimental groups shown in **Figures 3A,B** (*group* × *stimulus* × *trial* GLM for repeated measures; *group effect*: *F*_1,88_ = 1.1, NS), thus we pooled their results in a single graphic (**Figure 3C**). Statistical effects in this pooled group (**Figure 3C**) were equivalent to those described for experimental groups presented in **Figures 3A,B** both during acquisition (*stimulus* × *trial interaction: F*_18,1584_ = 11.2, *p* < 0.001; Tukey test; *stimulus* × *trial 10 effect*; A+ vs. B+ and A+ vs. AB-: *p* < 0.01; B+ vs. AB-: *p* < 0.001) and retention tests (GLM for repeated measures; *F*_2,178_ = 37.0, *p* < 0.001; Tukey test; A+ vs. B+ and A+ vs. AB-: *p* < 0.01; B+ vs. AB-: *p* < 0.001). In conclusion, while an ITI of 8 min allowed solving both PP (**Figure 2**) and NP (**Figure 3**), only PP could be solved with a shorter ITI of 3 min (**Figure 1**).

### Distinct Learning Categories in Bimodal Patterning Solving

Considering that responses to element and compound stimuli during retention tests reflected very well the level of discrimination reached in bimodal PP (**Figure 2**) or NP (**Figure 3**), we analyzed these responses at the individual level to classify bees into distinct learning categories. In retention tests, bees could respond or not only once to each of the three stimuli (A, B, and AB), thus eight different combinations of response may emerge (000, 111, 100, 010, 001, 011, 110, 101; to A, B, and AB, respectively). Successful learners of a PP task should not respond to the unrewarded A and B elements, and respond to the rewarded compound AB (001). Successful learners of NP should respond to the rewarded elements A and B, and not respond to the unrewarded compound AB (110). We thus asked: what is the proportion of bees presenting successful performances in retention tests after bimodal PP and NP? Which are the other categories of response emerging during these tasks? How do the learning curves of bees in these different categories look like? To answer these questions we analyzed the individual response of 90 bees trained to bimodal PP (**Figure 2C**) or NP (**Figure 3C**) with an ITI of 8 min.

**FIGURE 4:**
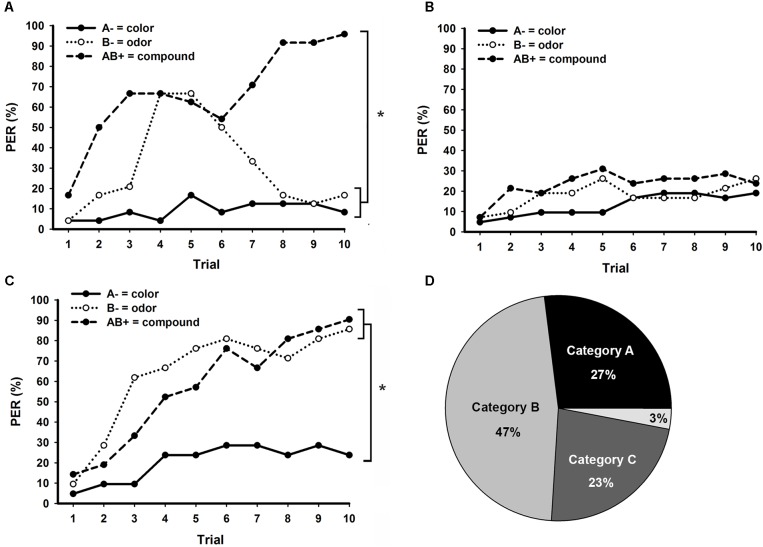
Distinct learning categories observed in bees trained to bimodal positive patterning (PP) with an ITI of 8 min. Conditioning consisted of 10 trials of each stimulus presented in a pseudorandom sequence. **(A)** Performance of bees that presented exact correct responses during retention tests (good PP learners), non-responding to the unimodal elements and responding to the bimodal compound (*N* = 24). **(B)** Performance of bees that responded or not to all three stimuli during retention tests, thus presenting a generalist strategy (*N* = 42). **(C)** Performance of bees that did not respond to the visual element A, but responded to the olfactory element B and the compound stimulus AB during retention tests (*N* = 21). **(D)** Percentage of bees in each of the PP learning categories presented in A (Category A), B (Category B) and C (Category C). Only 3% of the bees could not be classified into one of these three categories. Asterisks indicate significant differences in GLM followed by Tukey test comparing responses to each stimulus in the last conditioning trial.

**Figure 4** shows the three major learning categories emerging in bees trained to bimodal PP, classified according to their responses in retention tests. From 90 bees (**Figure 2C**), only 24 (27%) presented exactly correct responses (001) in retention tests (**Figure 4A**). Surprisingly, almost half of the bees (*n* = 42; 47%) responded equally to all three stimuli (000 or 111), thus presenting a generalist strategy toward unimodal elements and compound stimuli (**Figure 4B**). The third major category of response (*n* = 21; 23%) emerging during bimodal PP solving consisted of individuals not responding to the unrewarded visual element A, but responding to the unrewarded olfactory element B and the compound stimulus AB (011; **Figure 4C**). Only three bees (3%) could not be classified in one of these three learning categories (**Figure 4D**). Two of them responded only to the olfactory element B (010), whereas one bee responded to A and AB, but not to B (101).

Bees classified as good PP learners (**Figure 4A**) clearly solved the task, but as observed in the overall performance of bees trained to PP (**Figure 2C**), they presented increasing responses to the unrewarded odor (B) at the beginning and around the fifth trial started to suppress these responses. At the end of conditioning, these bees noticeably discriminate between each unrewarded unimodal element and the rewarded bimodal compound (*stimulus* × *trial* GLM for repeated measures; *interaction: F*_18,414_ = 9.3, *p* < 0.001; Tukey test; *stimulus* × *trial 10 effect*; A- vs. B-: NS; A- vs. AB+, and B- vs. AB+: *p* < 0.001). On the other hand, bees presenting generalist responses (**Figure 4B**) were completely unable to discriminate between any of the stimuli during bimodal PP (*stimulus* × *trial* GLM for repeated measures; *interaction: F*_18,738_ = 1.4, NS). The last learning category observed in bimodal PP (**Figure 4C**) was composed by bees that discriminate between the visual element A and the other stimuli, but could not differentiate the olfactory element B from the bimodal compound AB (*stimulus* × *trial* GLM for repeated measures; *interaction: F*_18,342_ = 2.6, *p* < 0.001; Tukey test; *stimulus* × *trial 10 effect*; A- vs. B- and A- vs. AB+: NS; B- vs. AB+: NS).

**FIGURE 5:**
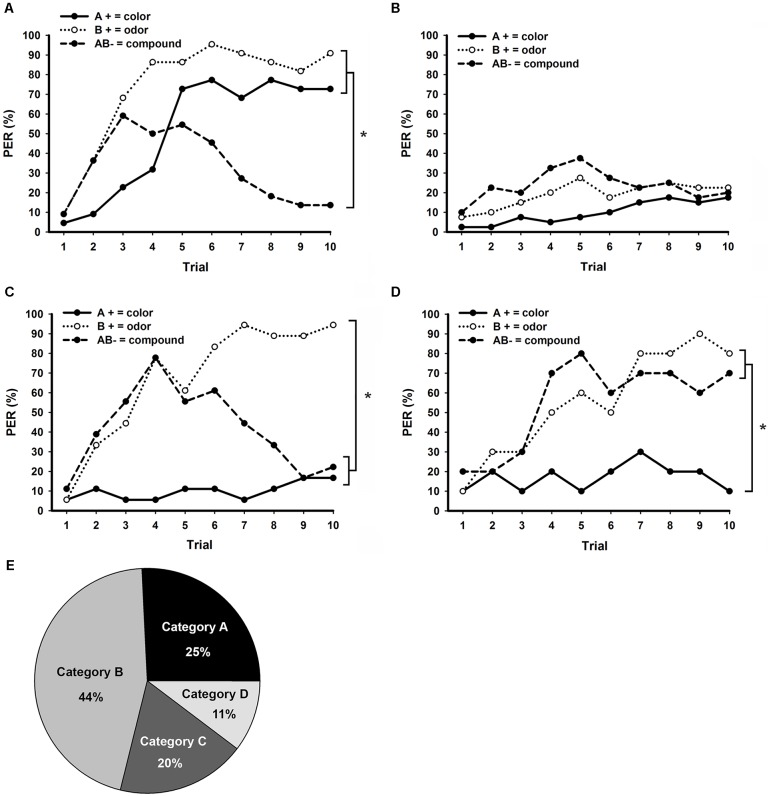
Distinct learning categories observed in bees trained to bimodal negative patterning (NP) with an ITI of 8 min. Conditioning consisted of 10 trials of each stimulus presented in a pseudorandom sequence. **(A)** Performance of bees that presented exact correct responses during retention tests (good NP learners), responding to the unimodal elements and non-responding to the bimodal compound (*N* = 22). **(B)** Performance of bees that responded or not to all three stimuli during retention tests, thus presenting a generalist strategy (*N* = 40). **(C)** Performance of bees that only responded to the olfactory element B during retention tests (*N* = 18). **(D)** Performance of bees that responded to the olfactory element B and the bimodal compound AB, but not to the visual element A during retention tests (*N* = 10). **(E)** Percentage of bees in each of the PP learning categories presented in A (Category A), B (Category B), C (Category C), and D (Category D). Asterisks indicate significant differences in GLM followed by Tukey test comparing responses to each stimulus in the last conditioning trial.

All 90 bees trained to bimodal NP (**Figure 3C**) could be classified into one of the following four learning categories according to their responses in retention tests: good NP learners (110; **Figure 5A**); generalists (000 or 111; **Figure 5B**); responding only to B (010; **Figure 5C**); responding to B and AB (011, **Figure 5D**). Bees classified as good NP learners (**Figure 5E**; *N* = 22; 25%) were clearly able to discriminate between each rewarded unimodal element and the unrewarded bimodal compound (**Figure 5A**; *stimulus* × *trial* GLM for repeated measures; *interaction:* F_18,378_ = 7.0, *p* < 0.001; Tukey test; *stimulus* × *trial 10 effect*; A+ vs. B+: NS; A+ vs. AB-, and B+ vs. AB-: *p* < 0.001). As well as in bimodal PP (**Figure 4B**), a large amount of bees confronted to a bimodal NP task (**Figure 5E**; *N* = 40, 44%) developed a generalist strategy and were totally unable to discriminate between the three stimuli (**Figure 5B**; *stimulus* × *trial* GLM for repeated measures; *interaction: F*_18,702_ = 1.5, NS).

The third most representative learning category observed in bimodal NP (**Figure 5E**; *N* = 18; 20%) was composed by bees that discriminate between the olfactory element B and the other stimuli, but could not differentiate the visual element A from the bimodal compound AB (**Figure 5C**; *stimulus* × *trial* GLM for repeated measures; *interaction: F*_18,306_ = 6.7, *p* < 0.001; Tukey test; *stimulus* × *trial 10 effect*; A+ vs. B+ and B+ vs. AB-: *p* < 0.001; A+ vs. AB-: NS). Finally, the fourth learning category emerging in bimodal NP (**Figure 5E**; *N* = 10; 11%) was composed by bees that presented low response levels to the rewarded visual element A, but developed increasing responses to the rewarded element B and the unrewarded compound AB (**Figure 5D**; *stimulus* × *trial* GLM for repeated measures; *interaction: F*_18,162_ = 1.9, *p* < 0.05; Tukey test; *stimulus* × *trial 10 effect*; A+ vs. B+ and A+ vs. AB-: *p* < 0.001; B+ vs. AB-: NS).

## Discussion

Our study shows that honey bees are able to solve bimodal PP and NP in a classical PER conditioning protocol using a colored-light screen as visual stimulus and a pure synthetic odor as olfactory stimulus. The two pairs of visual-olfactory stimuli used in our experiments (green 535 nm/2-hexanol or blue 455 nm/1-nonanol) induced equivalent levels of bimodal patterning discrimination, both in PP and NP. While an ITI of 8 min allowed solving both PP and NP tasks, only PP could be solved with a shorter ITI of 3 min. This result agrees with previous experiments on olfactory patterning discrimination that found better performances using longer trial-spacing (Deisig et al., 2007). More precisely, these authors found that honeybees trained in olfactory PER conditioning were unable to solve PP or NP with ITIs of 1 min or 3 min, whilst an ITI of 5 min allowed solving only PP. As well as in our bimodal patterning approach, an ITI of 8 min favored solving of both olfactory PP and NP (Deisig et al., 2007). Many reasons can account for the fact that an ITI of 3 min allowed bimodal PP solving in our work, but not olfactory PP solving in that previous study: nature of the stimuli (visual-olfactory vs. only olfactory); number of trials (10 per stimulus vs. 4 per element and 8 for the compound); duration of CS presentation (7 s vs. 6 s); experimental context (dark room and 45% body inclination vs. illuminated room and vertical body position), among others. Altogether, results on trial-spacing effect in patterning solving by bees are in line with an extensive literature showing that animals often present better learning when CS trials are temporally more spaced (Gibbon et al., 1977; Barela, 1999; Sunsay et al., 2004).

Previous studies in olfactory patterning discrimination by bees suggest that a balanced proportion of reinforced and non-reinforced trials (1:1 CS+ /CS- rate) favors discrimination in those tasks (Deisig et al., 2001, 2007). In the new bimodal conditioning approach here developed, we had an important limitation to develop PP and NP with such a contingency balance: reasonable levels of visual learning by harnessed bees are only reached with a large amount of trials (Avarguès-Weber and Mota, 2016). If we performed 10 trials for each element and 20 trials for the bimodal compound (1:1 contingency rate) with an ITI of 8 min, our conditioning protocol together with the memory test would least more than six hours. Considering also the time for capturing and harnessing the bees, as well as the one hour resting period prior to conditioning, it was simply impossible for us to perform such an experiment. We actually tried to perform a protocol using five trials of each element and 10 trials of the compound with an ITI of 8 min, but the levels of learning obtained for the visual element were very poor and there was no successful discrimination in PP or NP (data not shown).

The fact that only a bimodal PP task could be solved with a shorter ITI of 3 min, but not a NP task is in agreement with several studies observing that PP may be learned using a different strategy than NP (e.g. Rescorla, 1972; Bellingham et al., 1985; Kehoe, 1988; Deisig et al., 2007). Solving of NP tasks in rabbits requires longer CS and ITI duration than PP tasks (Kehoe and Graham, 1988; Kinder and Lachnit, 2002). In humans, longer processing time was found in response to the compound stimulus during NP when compared to PP (Lachnit et al., 2002). Interestingly, olfactory NP solving in bees requires olfactory input from both the antennae, whereas PP can be solved with unilateral olfactory stimulation of a single antennae (Komischke et al., 2003). Together with our data, these results in different models support the assumption that NP is solved using a learning strategy that requires different resources than the ones employed to solve PP.

It has been suggested that PP solving admits an *elemental learning* strategy, whereas NP solving can exclusively rely in a *configural learning* (non-elemental) strategy (Pearce, 1994; Deisig et al., 2001; Giurfa, 2003; Devaud et al., 2015). In the present work, we analyzed the individual performances of 90 honey bees in bimodal PP or NP, and we found that different learning strategies emerged in both these paradigms (see section “Distinct learning categories in bimodal patterning solving”). The first surprising observation from this analysis was the high percentage of bees that presented strong generalization between all stimuli and were thus unable to solve the discrimination both in the PP and the NP approach (47 and 44% of bees, respectively). These results highlight the level of difficulty of these tasks and rule out the possibility that bees solve them using an *extreme configural learning* strategy (Williams and Braker, 1999). These *extreme configural* theories are different from Pearce’s *configural* theory (Pearce, 1994), because they predict no generalization between a compound and its elements, since the compound would be treated as a totally new stimulus completely unrelated to its elements (Williams and Braker, 1999; Deisig et al., 2003).

Apart from bees that completely generalized between stimuli in PP or NP, we also found intermediate learning categories that presented generalization between one of the elements and the compound. In the case of PP, generalization between the olfactory element and the compound occurred in 23% of bees. Bees in this learning category seemed to reduce the complexity of the problem by treating it as an elemental differential conditioning task (A- vs. AB/B+). The olfactory element B and the compound AB appeared to be both treated as one rewarded odor. In the case of NP, generalization between one of the elements and the bimodal compound was found not only for the olfactory element B as in the bimodal PP task, but also for the visual element A. Curiously, a representative amount of individuals trained to bimodal NP (20%) generalized between the visual element A and the compound AB, as if they solved the task following a differential conditioning schedule (A/AB- vs. B+). Furthermore, a smaller amount of bees (11%) trained to NP, developed generalized responses between the olfactory stimulus B and the compound AB that were similar to the ones observed in some bees trained to PP (A- vs. AB/B+). In this case, however, bees were twice wrong in their responses, because they were supposed to respond to A+ and not respond to AB- in the NP task. Although different categories of generalization emerged in bimodal PP (to all stimuli; between odor and compound) and NP (to all stimuli; between odor and compound; between color and compound), the equivalent total amount of unsuccessful bees in these tasks (63 and 65%, respectively) suggests a similar difficulty for bees to solve them.

Although most of the bees trained to bimodal PP or NP task presented generalization and were unsuccessful in solving the task, we also identified a category of very efficient learners in both these approaches (27 and 25% of bees, respectively). Our results, therefore, reinforce the notion that averaged learning curves and memory retention scores obtained from a group of animals often hide a more elaborate range of learning dynamics that can only be observed at the individual level (Gallistel et al., 2004). Accordingly, several recent studies on learning and memory by bees emphasize the importance of better analyzing the dynamics of individual performances (e.g., Mota and Giurfa, 2010; Dobrin and Fahrbach, 2012; Pamir et al., 2014; Evans et al., 2017; Vieira et al., 2018). Similar to our results on bimodal patterning, only 30% of 111 bees trained to a multiple olfactory reversal task were able to accurately solve this non-elemental problem (Mota and Giurfa, 2010). An extensive evaluation of individual performances of 3298 bees in different elemental olfactory tasks also revealed that group-averaged learning analysis hid drastic inter-individual differences (Pamir et al., 2014). Altogether, these studies indicate that finding a single associative learning theory to explain the averaged performance of a population is tricky, and may not reflect the real complexity of CS and US representations at the individual level. Effective learners of bimodal PP may use *elemental* or *configural* strategies to solve this task, whereas good learners of bimodal NP could only use *configural* strategies. Finding which of those alternative accounts is actually used by bees remains so far a challenge, even for the more studied unimodal olfactory PP and NP in honey bees (Deisig et al., 2003, 2007; Devaud et al., 2015).

Although individual bees trained to elemental olfactory tasks differ in terms of the number of trials required to develop the first conditioned response (CR), as well as the stability of the CR along trials, high levels of success are usually reached at the end of conditioning (Pamir et al., 2014). Pamir and collaborators found that 54% of the responsive animals trained to elemental olfactory tasks already developed conditioned responses (CR) to the CS+ after a single trial of conditioning. By the third trial about 80% of those animals presented correct responses to the CS+. They also show a high average level of CR stability once the animal starts to respond. The average percentage of non-responding animals in elemental olfactory tasks was only ∼20%. All in all, this analysis reveals high levels of individual success (∼80%) on the solving of elemental forms of associative learning by harnessed bees (Pamir et al., 2014). On the other hand, only 25 to 30% of harnessed bees were able to solve a non-elemental olfactory task (Mota and Giurfa, 2010) or a bimodal patterning discrimination (present study). These studies indicate that honey bees present much higher rates of success in solving elemental than non-elemental tasks in a classical conditioning framework. Moreover, the equivalent low levels of success obtained in bimodal PP and NP tasks in our study suggests that PP is probably solved by bees using an non-elemental (*configural*) learning strategy.

Previous studies in non-human mammals suggested that the use of different sensory modalities in patterning tasks may favor the emergence of *elemental* rather than *configural* strategies (e.g., Redhead and Pearce, 1995; Brandon et al., 2000; Myers et al., 2001). The relative salience of the elements is also an important feature that can influence the associations acquired in patterning discriminations, particularly in NP tasks (Delamater, 2012). For instance, when stimuli with different saliences were used in NP tasks, discrimination was first learned between the unrewarded compound and the less salient element, as compared with the more salient element (Redhead and Pearce, 1995; Delamater et al., 1999). Studies on bimodal learning by honey bees have often suggested that odors are more salient cues than colors (e.g. Couvillon and Bitterman, 1989; Funayama et al., 1995; Greggers and Mauelshagen, 1997), especially in harnessed individuals (Gerber and Smith, 1998; Mota et al., 2011). Probably for that reason, learning levels acquired in visual PER conditioning are typically lower than those reported for olfactory PER conditioning (Avarguès-Weber and Mota, 2016), as also observed in the present study. The higher salience of odors over colors in our bimodal patterning approach may be responsible for the strong generalization observed between the olfactory element B and the compound AB, as well as the better levels of discrimination between the visual element A and the compound AB, in certain cases.

A recent study showed that the mushroom bodies (MBs) of the honey bee brain are necessary for solving both olfactory PP and NP (Devaud et al., 2015). Pharmacological inhibition of the MBs disrupted the capacity of bees to solve PP and NP, but not their ability to learn elemental olfactory discriminations. Therefore, apart from the well-known role of the MBs in memory storage and retrieval, theses insect brain structures seem to be implicated in the acquisition of ambiguous olfactory discrimination problems (Devaud et al., 2015). The necessity of the MBs for solving olfactory PP and NP, but not elemental olfactory discriminations, strongly suggests that olfactory PP is solved by bees using a non-elemental rather than an elemental summation strategy (Devaud et al., 2015). Little is known, however, about the role of the MBs on elemental and non-elemental visual or bimodal learning in bees. It might be that the simple necessity of integrating visual and olfactory information for bimodal patterning learning would require the integrative role of the MBs.

The MBs are indeed the main region of the honey bee brain where a convergence between visual and olfactory neural circuits was clearly identified (Mobbs, 1982; Ehmer and Gronenberg, 2002). Considering that a cross-modal interaction between olfactory and visual cues is necessary to solve bimodal PP and NP, the MBs appear as the most probable structures mediating these discriminations. A recent study in *Drosophila* found that visual and olfactory associative learning share dopaminergic neural circuits in the MBs, confirming that distinct sensory memories are processed in this common brain center (Vogt et al., 2014). Alternative regions for cross-talk between visual and olfactory circuits in the bee brain have also been suggested in the median, lateral, and posterior protocerebrum (Erber and Menzel, 1977; Maronde, 1991), but the role of these structures in learning and memory remains poorly understood. Future studies should combine the new bimodal PP and NP protocols here presented to pharmacological or neurophysiological techniques, in order to uncover the neural mechanisms underlying these cognitive phenomena.

## Author Contributions

TM conceived the study and designed the methodology. BM, JR, and TM performed the experiments and analyzed the data. BM and TM wrote the first draft of the manuscript. All authors contributed to the final version of the manuscript.

## Conflict of Interest Statement

The authors declare that the research was conducted in the absence of any commercial or financial relationships that could be construed as a potential conflict of interest.
